# Development and Validation of a Chromatography Method Using Tandem UV/Charged Aerosol Detector for Simultaneous Determination of Amlodipine Besylate and Olmesartan Medoxomil: Application to Drug-Excipient Compatibility Study

**DOI:** 10.1155/2017/4878316

**Published:** 2017-12-17

**Authors:** Ariadne M. Brondi, Jerusa S. Garcia, Marcello G. Trevisan

**Affiliations:** LACFar-Institute of Chemistry, Federal University of Alfenas (UNIFAL-MG), Alfenas, MG, Brazil

## Abstract

A study was carried out to investigate compatibility of amlodipine besylate and olmesartan medoxomil with a variety of pharmaceutical excipients. Both drugs are antihypertensive agents that can be administered alone, in monotherapy, or in pharmaceutical association. The studies were performed using binary and ternary mixtures, and samples were stored for 3 and 6 months at 40°C under 75% relative humidity and dry conditions. For this study, a method based on high-performance liquid chromatography (HPLC) was developed and validated for the simultaneous determination of amlodipine besylate and olmesartan medoxomil in samples from pharmaceutical preformulation studies using diode array detector (DAD) and charged aerosol detector (CAD). The runtime per sample was 10 min with retention time of 7.926 min and 4.408 min for amlodipine and olmesartan, respectively. The validation was performed according to ICH guidelines. The calibration curve presents linear dynamic range from 12 to 250 *μ*g mL^−1^ for amlodipine and from 25 to 500 *μ*g mL^−1^ for olmesartan with coefficient of determination (*R*^2^ ≥ 0.9908) while repeatability and reproducibility (expressed as relative standard deviation) were lower than 1.0%. The excipients such as corn starch, croscarmellose sodium, magnesium stearate, polyvinyl alcohol, talc, polyvinylpyrrolidone, lactose monohydrate, and polyethylene glycol showed potential incompatibilities after accelerated stability testing.

## 1. Introduction

**Figure 1:**
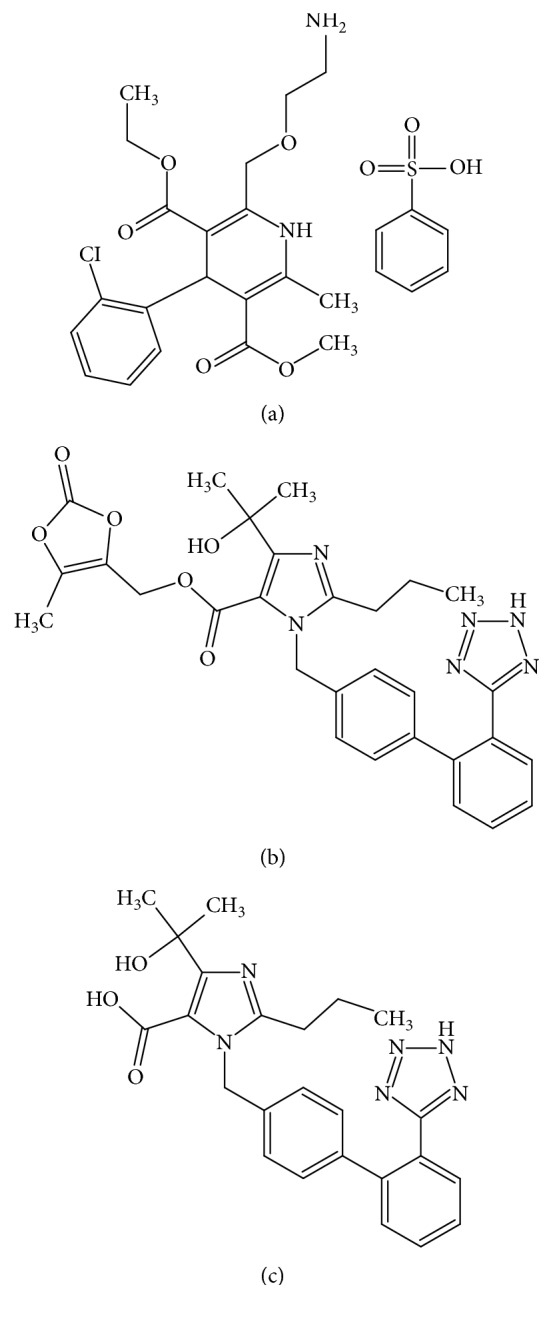
Chemical structure of (a) amlodipine besylate, (b) olmesartan medoxomil, and (c) olmesartan.

Amlodipine besylate (AMLO), CAS = 111470-99-6, 3-ethyl-5-methyl-2-(2-aminoethoxymethyl)-4-(2-chlorophenyl)-1,4-dihydro-6-methyl-3,5-pyridinedicarboxylate benzenesulphonate, is a calcium channel blocker used in treatment of hypertension, angina pectoris, and other cardiovascular diseases.  Figure 1(a) shows the chemical structure of amlodipine besylate [1–3].

Olmesartan medoxomil (OLME), CAS = 144689-63-4, 1H-imidazole-5-carboxylic acid, 4-(1-hydroxy-1-methylethyl)-2-propyl-1-[[2′-(1H-tetrazol-5-yl)[1,1′-biphenyl]-4yl]methyl]-, (5-methyl-2-oxo-1,3-dioxol-4-yl) methyl ester, is a prodrug that is subjected to enzymatic hydrolysis, and its active product, olmesartan, is an angiotensin II receptor blocker (ARB) used in treatment and prophylaxis of hypertension. Figures 1(b) and 1(c) show the chemical structure of olmesartan medoxomil and of its active product, olmesartan [4–7].

At the treatment of hypertension, the monotherapy has adequate therapeutic response, but at least 70% of the patients require two or more medications for the adequate blood pressure control. For these cases, the pharmaceutical association is a proper administration of drugs [8].

The United States Pharmacopeial Convention (USP) recommends for the determination of AMLO the liquid chromatography (LC) assay using a mobile phase containing methanol, acetonitrile, and buffer solution (trimethylamine and phosphoric acid, pH 3.0) at the proportion of 35 : 15 : 50. For the determination of OLME, the USP recommends the LC assay using a mobile phase containing buffer solution (phosphoric acid, pH 3.4) and acetonitrile at the proportion of 33 : 77 [9]. The simultaneous determination of AMLO and OLME is already reported in the literature in human plasma [2, 10], urine, [10] and dosage form [11–13]. However, to date, there are no reports on the use of charged aerosol detector (CAD) on determination of AMLO and/or OLME.

The CAD is a relatively recent technology, and its main characteristic is to be a universal detector for nonvolatile analytes. The response may vary according to the composition of the mobile phase, and there are some limitations about the mobile phase: these may not be saline and the solvent must be volatile. The CAD is a detector independent of the chemical structure, optical properties, and the ionization capacity of the analytes, producing a similar response for different compounds, which allows the quantification even when there is no standard availability, since the detector measures the amount of charge, which is proportional to the amount of analytes. At CAD, the HPLC eluent is nebulized by Venturi effect produced by a carrier inert gas, usually nitrogen, transforming the eluent into small droplets. The small droplets are carried by the gas flow to a drying tube where the solvent is evaporated. The aerosol formed is charged with a secondary stream of nitrogen that has passed a high-voltage platinum wire, and the charged aerosol is measured by an electrometer [14–21].

The drug-excipient compatibility study is an important step in the development of pharmaceutical dosage forms. The incompatibility of a drug with one or more excipients in a formulation can affect the stability and/or bioavailability of the active product, thereby affecting its safety and efficacy of the formulation. The drug-excipient incompatibility can be due to the covalent reaction between the drug and the excipient, and/or the excipient-promoted intrinsic degradation of the drug. This study also allows to know the type of packaging most suitable and to establish adequate conditions of storage [22–25].

The purpose of this work was to develop and validate a sensitive LC method with CAD for determination of AMLO and OLME, which could be applied to drug-excipient compatibility studies.

## 2. Experimental

### 2.1. Chemicals and Reagents

Amlodipine besylate and olmesartan medoxomil were supplied by Aché (Guarulhos, Brazil). The pharmaceutical excipients, such as corn starch, microcrystalline cellulose, croscarmellose sodium, magnesium stearate, titanium dioxide, polyvinyl alcohol, talc, polyvinylpyrrolidone (PVP), lactose monohydrate, and polyethylene glycol (PEG), generally used in the formulation with AMLO and OLME, were of USP grade. Acetonitrile of LC grade (J.T. Baker, USA) and extrapure ammonium acetate (Nuclear, Brazil) were used to prepare the mobile phase for LC analysis. For development and validation of HPLC-CAD method, amlodipine besylate pharmaceutical standard (Sigma-Aldrich, Saint Louis, USA) and olmesartan medoxomil European Pharmacopoeia Reference Standard (European Directorate for the Quality of Medicines & Healthcare, Strasbourg, France) were used. A Master System MS2000 water purification (Gehaka, Brazil) was used to obtain ultrapure water (resistivity of 18 MΩ cm^−1^).

### 2.2. Apparatus and Chromatographic Conditions

For HPLC analysis, liquid chromatographic system UHPLC Ultimate 3000 (Thermo Scientific Dionex, USA), consisting of an LPG-3400RS pump with integrated vacuum degasser, autosampler WPS-3000RS with 100 *μ*L injector, TCC-3000RS column oven, DAD-3000RS diode array detector, and Dionex Corona ultra RS charged aerosol detector, was used. In brief, isocratic elution with the column Eclipse XDB-C18 (4.6 × 250 mm, 5 *μ*m; Agilent, Saint Clair, USA) at room temperature (25°C) employing a mobile phase consisting of ammonium acetate (pH 6.8 at 0.5 mol L^−1^) and acetonitrile (40 : 60, v/v) was carried out. The injection volume was 10 µL, and the flow rate was 0.5 mL min^−1^. Mobile phase, standard solutions, and samples were filtered through nylon filter (0.45 µm; Sartorius, Göttingen, Germany) before injection into the chromatographic system. The software Chromeleon 6.8 (Thermo Scientific Dionex, Waltham, USA) was used to record chromatograms, peak quantification, and integration. This methodology was inspired by the methodology described by Qutab et al. [12]. The change in flow rate was performed to improve the chromatographic resolution and symmetry of the peaks, and the reduction of injection volume occurred due to the high concentrations of samples to be determined.

The optimum conditions of CAD used the acquisition rate was 10 Hz to have a sufficient number of data points, and the time constant filter, used to reduce the noise in chromatogram, was level 0. The optimization of CAD conditions was performed observing the resolution and symmetry of the peaks.

### 2.3. Validation of the Method

Validation studies were performed according to ICH guidelines to characterize the proposed analytical method such as dynamic range, calibration curve, limit of detection (LOD), limit of quantitation (LOQ), accuracy, precision, and method robustness [26].

The dynamic range was selected within 15.0–250.0 *μ*g mL^−1^ for AMLO and 25.0–500.0 *μ*g mL^−1^ for OLME. Linear and polynomial (order 2) calibration curves were obtained by plotting the peak area against the nominal concentration by least squares [22, 27].

To determine LOD and LOQ, comparisons between measurements of reference standard solutions at low concentrations of analyst and blank samples were performed; the LOD and LOQ were defined as signal-to-noise ratio of 3 : 1 and 10 : 1, respectively [26, 28].

The accuracy was determined by the recovery method. Three samples were analyzed before and after the addition of a known amount of each analyte standard solution. The precision indicates the ability of the methodology to provide reproducible results. The intraday precision was evaluated by replicates of three samples on one day, whereas the interday precision was determined over three consecutive days.

Specificity was determined by comparing any interfering peaks from the blank, pure excipients, and ternary mixtures at initial time injection and checking the peak purity by HPLC-DAD.

Small changes in chromatographic conditions such as mobile phase (proportion of acetonitrile in 60 ± 2%), column temperature (25 ± 5°C), and flow rate (0.5 ± 0.1 mL min^−1^) were done to evaluate the robustness of the proposed HPLC method.

### 2.3. Drug-Excipient Compatibility Studies

The stability study was performed using AMLO and OLME alone, binary mixtures formed by AMLO and each selected pharmaceutical excipient at a 1 : 1 (m/m) ratio, binary mixtures formed by OLME and each selected pharmaceutical excipient at a 1 : 1 (m/m) ratio, ternary mixtures formed by AMLO, OLME, and each selected pharmaceutical excipient at a 1 : 4 : 5 (m/m/m) ratio, and a binary mixture formed by AMLO and OLME at a 1 : 4 (m/m) ratio. The mixtures were prepared using analytical balance (Shimadzu AUW220D) and submitted to a physical homogenization using a vortex for 5 min. Each mixture was quartered into five portions. The first portion of samples was analyzed immediately. Two portions of samples were also stored for 3 and 6 months in a stability chamber at 40 ± 0.5°C using a saturated NaCl solution (average of 75 ± 1% RH) monitored by a temperature/humidity data logger. And, two portions of samples were also stored for 3 and 6 months in a stability chamber at 40 ± 0.5°C and dry conditions [22].

## 3. Results and Discussion

### 3.1. Method Validation

**Figure 2:**
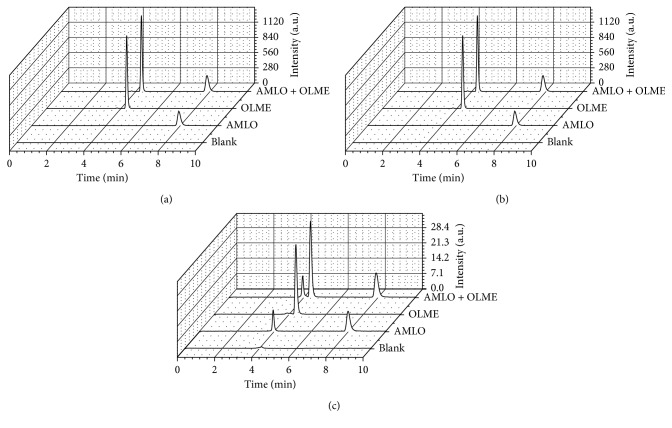
Chromatograms recorded at (a) 239 nm, (b) 250 nm, and (c) CAD.

The chromatographic results were evaluated using DAD and CAD. At DAD, the optimum wavelengths selected were 239 nm and 250 nm for AMLO and OLME, respectively. The typical chromatograms of AMLO and OLME obtained by the HPLC-based method are presented in Figure 2. The average retention times of OLME and AMLO were 4.336 and 7.854 min, respectively. In the AMLO chromatogram obtained by CAD, there are two peaks: the first at 3.987 min, from besylate fraction, and the second peak at 7.926 min, from AMLO. The OLME chromatogram registered by CAD present only one peak at 4.408 min. The delay observed at retention time registered by DAD and CAD occurs because of the additional distance that the flow must travel to reach this detector.

#### 3.1.1. Calibration Curve

The dynamic range and calibration curve were estimated by analyzing AMLO and OLME standards. The calibration curves of AMLO and OLME were built at the range from 15.0 to 250.0 *μ*g mL^−1^ and from 25.0 to 500.0 *μ*g mL^−1^, respectively. The calibration curves were linear with DAD response. The coefficient of determination (*R*^2^) was equal to or better than 0.995. The relative standard deviation values of the slope were equal to or better than 3%. For each point of calibration standards, the concentrations were recalculated from the equation of the linear regression curves. The calibration curves with CAD data were calculated with linear and polynomial fits (Table 1). The polynomial fit showed better coefficient of determination (*R*^2^ > 0.999) than linear fit (*R*^2^ > 0.991). A linear response was not expected, since aerosol charging does not depend directly on the aerosol mass. Although it is known that Corona CAD response is nonlinear within the range of four orders of magnitude, we found that the signal is nearly linear in the examined ranges of analyzed compounds [14–21, 29].

#### 3.1.2. Limit of Detection (LOD) and the Limit of Quantification (LOQ)

Determination of the signal-to-noise ratio (S/N) was performed by comparing the measured signals from samples of known low concentrations of the analyte with those of blank samples and establishing the minimum concentration at which the analyte can be reliably detected. The LOD and LOQ for each calibration curve are presented in Table 1.

#### 3.1.3. Accuracy and Precision

The accuracy of the recovery for AMLO and OLME was evaluated at three concentrations. The mean recoveries for all samples from each run were in the range of 95.46–101.85% (Table 2). The precisions were calculated from ten consecutive injections of a sample, and the observed RSD values for AMLO and OLME were in the range of 0.42–0.57%. Intermediate precision was calculated from three days, with RSD values in the range of 0.67–0.96%.

#### 3.1.4. Specificity

**Figure 3:**
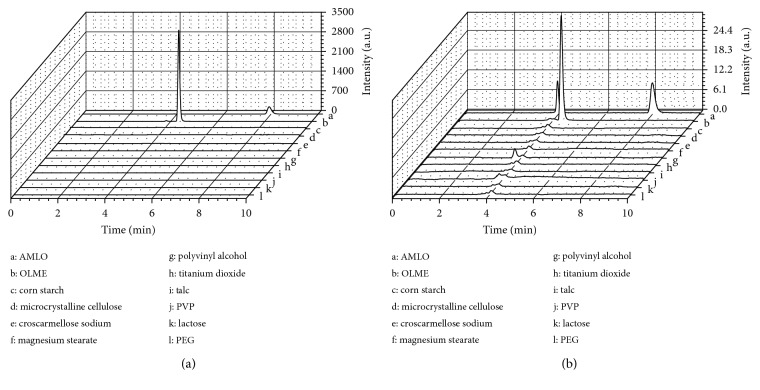
Chromatograms of pure drugs and excipients by (a) DAD and (b) CAD.

**Figure 4:**
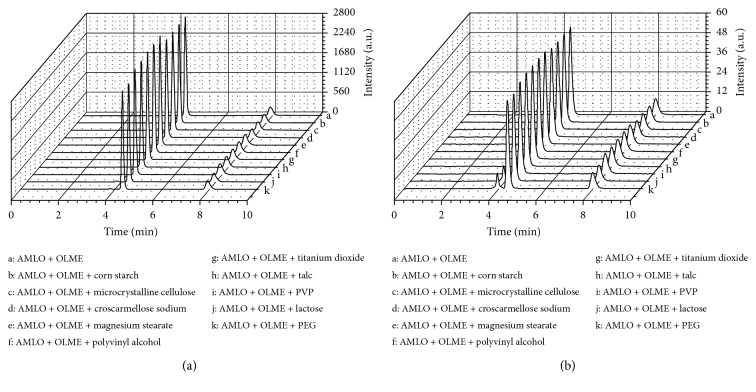
Chromatograms of ternary mixtures at initial time recorded by (a) DAD and (b) CAD.

The evaluation of the specificity of the method can be observed in Figures 2, 3, and 4. Figure 2 shows that the drugs have well-resolved peaks. Observing Figure 3, it can be stated that the excipients do not have peaks in the same retention time as drugs.  Figure 4 shows that even in the presence of the excipients, the drug peaks showed no change in retention times and symmetry. The chromatograms show that no significant interfering peaks were observed at the retention times of analytes, and the peaks are well resolved, with resolution > 1.5. The peak purity match, calculated by software Chromeleon at ternary mixtures assays, was 99.93% with 0.06% RSD for AMLO and 98.76% with 0.08% RSD for OLME, based on DAD spectra of each run.

#### 3.1.5. Robustness

The robustness of the proposed HPLC method was evaluated by slight changes of the chromatographic parameters including the flow rate (±0.1 mL min^−1^), mobile phase (proportion of acetonitrile in 60 ± 2%), and column temperature (±5°C). Afterwards, the drug content and retention time were determined. The results summarized in Table 3 demonstrated that the proposed HPLC method was robust for its intended applications.

### 3.2. Drug-Excipient Compatibility Study

The chromatograms of AMLO, OLME, and ternary mixtures with excipients at the initial time are presented in Figure 4. In both HPLC detectors, DAD and CAD, none of excipients tested showed interference on the chromatographic profile of the analytes, which makes the methodology adequate for the drug-excipient compatibility study.

After 3 and 6 months of incubation in a stability chamber at 40°C ± 1°C under dry and 75% ± 5% relative humidity conditions, the samples were analyzed to verify the content of AMLO and OLME. The samples were prepared so that the expected concentration of AMLO was 100 *μ*g mL^−1^ and OLME was 400 *μ*g mL^−1^, so these values corresponded to 100% of AMLO and OLME. The reduction of 5% in these values is indicative of drug degradation, and consequently, chemical incompatibility between the drug and the excipient [22]. The results of drug-excipient compatibility studies are summarized in Tables 4 and 5, obtained by chromatograms of binary mixtures [30].

At 40°C ± 1°C under dry conditions, AMLO and OLME, after 3 and 6 months, did not present changes in concentration recovery in binary and ternary mixtures, indicating that in dry conditions, the AMLO and OLME are compatible with the excipients in the studies.

At 40°C ± 1°C under 75% ± 5% relative humidity, after 3 months, AMLO and OLME did not present changes in concentrations in binary and ternary mixtures. However, after 6 months, the binary mixtures of AMLO with corn starch and lactose monohydrate and of OLME with PVP and lactose monohydrate presented concentration reduction. And, the ternary mixtures showed concentration reduction of AMLO and OLME in the presence of croscarmellose sodium, magnesium stearate, polyvinyl alcohol, talc, PVP, lactose monohydrate, and PEG. The ternary mixture containing corn starch showed concentration reduction only for AMLO. Because of these reductions in concentration of drugs, these excipients are not indicated for preparation of formulations containing AMLO and OLME. Thus, if any of these excipients that presented incompatibility are used in the formulation of AMLO and OLME, this should be kept away from humidity and at milder temperatures.

## 4. Conclusions

The HPLC method for simultaneous determination of AMLO and OLME was validated according to ICH guidelines. The Corona CAD detector was found to be a powerful tool and, because it is relatively recent, has few validated methodologies.

The DAD presents lower LOD and LOQ than CAD. However, both detectors present satisfactory accuracy, precision, specificity, and robustness. The principal advantage of CAD over DAD is the fact that it is a detector independent of the chemical structure and optical properties. CAD allows the quantification even when there is no standard availability because it produces similar response for different compounds, since the detector measures the amount of charge, which is proportional to the amount of analyte [14, 15].

The drug-excipient compatibility study showed that at 40°C, under dry conditions, there is no incompatibility, indicating that the tested excipients can be used at formulations containing AMLO and OLME, provided they are stored away from humidity. At 40°C, under 75% of relative humidity, the excipients such as corn starch, croscarmellose sodium, magnesium stearate, polyvinyl alcohol, talc, PVP, lactose monohydrate, and PEG showed chemical incompatibility with AMLO and OLME, and because of it these excipients are not indicated for the preparation of formulations of AMLO and OLME. Thus, if they are used, the formulation should be kept away from humidity and at milder temperatures.

## Figures and Tables

**Table 1:** Linear and polynomial fit equations for calibration curves for AMLO and OLME determined by HPLC-DAD and HPLC-CAD.

	Range (*μ*g mL^−1^)	Equation	*R* ^2^	LOD (*μ*g mL^−1^)	LOQ (*μ*g mL^−1^)
AMLO	15–250	DAD-linear	0.9953	1	4
		*y* = 0.5862*x* + 1.4772			
		CAD-linear	0.0083	4	10
		*y* = 0.0221*x* − 0.0356			
		CAD-polynomial	0.9994	4	10
		*y* = −2.10^−5^*x*^2^ + 0.0271*x* + 0.1454			

OLME	25–500	DAD-linear	0.9958	1	10
		*y* = 0.6918*x* + 4.1201			
		CAD-linear	0.9908	5	15
		*y* = 0.0159*x* − 0.817			
		CAD-polynomial	0.9988	5	15
		*y* = −2.10^−5^*x*^2^ + 0.0257*x* + 0.2194			

**Table 2:** Precision and accuracy for AMLO and OLME determined by HPLC-DAD and HPLC-CAD.

	Concentration added (*μ*g mL^−1^)	DAD (*n* = 5)			CAD (*n* = 5)		
		Recovery (%)	RSD intraday (%)	RSD interday (%)	Recovery (%)	RSD intraday (%)	RSD interday (%)
AMLO	20	95.46	0.54	0.96	95.52	0.57	0.88
	40	101.85	0.56	0.81	100.72	0.49	0.85
	80	100.31	0.42	0.68	99.45	0.51	0.91

OLME	50	99.34	0.48	0.89	99.22	0.52	0.93
	100	100.66	0.45	0.87	100.89	0.43	0.84
	150	97.92	0.39	0.67	99.72	0.42	0.89

*n*: number of replicates.

**Table 3:** Robustness study for AMLO and OLME determined by HPLC-DAD and HPLC-CAD.

Chromatographic condition	AMLO				OLME			
	DAD		CAD		DAD		CAD	
	Concentration (*μ*g mL^−1^)	*t*_*R*_ (min)	Concentration (*μ*g mL^−1^)	*t*_*R*_ (min)	Concentration (*μ*g mL^−1^)	*t*_*R*_ (min)	Concentration (*μ*g mL^−1^)	*t*_*R*_ (min)
Original method	176.4 ± 0.1	7.854	176.3 ± 0.1	7.926	300.3 ± 0.1	4.336	299.8 ± 0.1	3.987
Mobile phase: acetonitrile 62%	164.7 ± 0.1	7.556	165.3 ± 0.1	7.976	288.8 ± 0.1	4.327	287.5 ± 0.1	4.76
Mobile phase: acetonitrile 58%	165.5 ± 0.1	7.921	166.1 ± 0.1	8.341	309.2 ± 0.1	4.406	310.4 ± 0.1	4.755
Flow rate: 0.4 mL min^−1^	172.5 ± 0.1	8.291	171.4 ± 0.1	8.711	295.9 ± 0.1	4.512	294.7 ± 0.1	4.860
Flow rate: 0.6 mL min^−1^	173.1 ± 0.1	7.125	174.5 ± 0.1	7.545	298.2 ± 0.1	4.154	299.4 ± 0.1	4.500
Column temperature: 30°C	187.4 ± 0.1	7.601	188.6 ± 0.1	8.022	284.6 ± 0.1	4.391	285.4 ± 0.1	4.742
Column temperature: 20°C	181.3 ± 0.1	8.095	180.2 ± 0.1	8.514	280.1 ± 0.1	4.395	279.3 ± 0.1	4.745

*t*_*R*_: retention time.

**Table 4:** Recovery of AMLO and OLME after 3 and 6 months at 40°C ± 1°C under dry condition in binary and ternary mixtures.

Excipient	Binary mixtures				Ternary mixtures			
	3 months		6 months		3 months		6 months	
	AMLO (%)	OLME (%)	AMLO (%)	OLME (%)	AMLO (%)	OLME (%)	AMLO (%)	OLME (%)
Corn starch	102.9 ± 0.4	101.3 ± 0.5	98.2 ± 0.5	97.5 ± 0.6	99.4 ± 0.1	101.7 ± 0.6	98.0 ± 0.6	102.8 ± 0.9
	100.3 ± 0.2	101.7 ± 0.3	101.6 ± 0.6	98.7 ± 0.5	99.4 ± 0.6	100.6 ± 0.5	98.6 ± 0.9	103.6 ± 0.3
Microcrystalline cellulose	98.8 ± 0.5	98.4 ± 0.7	99.6 ± 0.5	97.5 ± 0.7	100.8 ± 0.6	102.3 ± 0.9	98.1 ± 0.8	103.7 ± 0.5
Croscarmellose sodium	109.2 ± 0.4	99.8 ± 0.3	97.9 ± 0.4	103.9 ± 0.5	101.1 ± 0.2	101.8 ± 0.6	98.9 ± 0.6	101.5 ± 0.4
Magnesium stearate	103.0 ± 0.5	98.0 ± 0.6	105.0 ± 0.7	100.9 ± 0.4	99.4 ± 0.6	99.0 ± 0.2	100.5 ± 0.4	100.9 ± 0.8
Polyvinyl alcohol	104.6 ± 0.4	98.0 ± 0.4	98.6 ± 0.5	102.5 ± 0.3	103.1 ± 0.4	100.2 ± 0.2	99.6 ± 0.3	101.9 ± 0.3
Titanium dioxide	104.3 ± 0.6	99.1 ± 0.2	101.2 ± 0.6	100.8 ± 0.6	99.8 ± 0.4	102.6 ± 0.8	99.8 ± 0.6	102.1 ± 0.7
Talc	98.0 ± 0.7	98.9 ± 0.6	98.2 ± 0.8	99.8 ± 0.8	103.7 ± 0.3	98.8 ± 0.7	99.5 ± 0.7	101.5 ± 0.6
PVP	101.1 ± 0.3	99.5 ± 0.5	100.8 ± 0.6	100.0 ± 0.5	98.5 ± 0.8	101.8 ± 0.8	101.9 ± 0.3	101.3 ± 0.9
Lactose monohydrate	100.2 ± 0.8	103.6 ± 0.9	97.9 ± 0.7	103.5 ± 0.8	98.5 ± 0.4	98.5 ± 0.2	100.5 ± 0.7	99.7 ± 0.5
PEG	99.3 ± 0.3	98.7 ± 0.4	101.4 ± 0.5	99.2 ± 0.5	102.3 ± 0.5	99.5 ± 0.2	99.3 ± 0.4	102.7 ± 0.5

**Table 5:** Recovery of AMLO and OLME after 3 and 6 months at 40°C ± 1°C under 75% ± 5% relative humidity in binary and ternary mixtures.

Excipient	Binary mixtures				Ternary mixtures			
	3 months		6 months		3 months		6 months	
	AMLO (%)	OLME (%)	AMLO (%)	OLME (%)	AMLO (%)	OLME (%)	AMLO (%)	OLME (%)
Corn starch	99.3 ± 0.5	102.4 ± 0.4	104.3 ± 0.2	107.1 ± 0.8	101.7 ± 0.4	99.2 ± 0.9	99.2 ± 0.5	110.1 ± 0.7
	99.0 ± 0.5	100.5 ± 0.7	74.2 ± 0.4	102.5 ± 0.8	100.5 ± 0.7	98.7 ± 0.9	82.7 ± 0.9	101.5 ± 0.7
Microcrystalline cellulose	102.3 ± 0.3	100.4 ± 0.4	98.2 ± 0.4	100.8 ± 0.5	105.9 ± 0.9	98.2 ± 0.4	101.7 ± 0.2	101.2 ± 0.2
Croscarmellose sodium	99.7 ± 0.8	100.3 ± 0.5	99.8 ± 0.3	102.6 ± 0.3	103.2 ± 0.8	99.4 ± 0.6	92.3 ± 0.3	93.7 ± 0.5
Magnesium stearate	100.8 ± 0.8	101.6 ± 0.4	99.6 ± 0.6	100.4 ± 0.6	99.0 ± 0.2	99.5 ± 0.4	81.2 ± 0.7	83.1 ± 0.7
Polyvinyl alcohol	98.3 ± 0.9	98.5 ± 0.9	100.7 ± 0.8	102.9 ± 0.4	98.2 ± 0.6	100.3 ± 0.2	84.4 ± 0.6	83.6 ± 0.2
Titanium dioxide	98.1 ± 0.9	100.6 ± 0.5	98.3 ± 0.4	99.9 ± 0.5	98.4 ± 0.6	98.1 ± 0.4	101.8 ± 0.3	104.3 ± 0.4
Talc	99.9 ± 0.7	98.7 ± 0.6	104.1 ± 0.5	101.1 ± 0.3	99.4 ± 0.3	100.5 ± 0.5	84.2 ± 0.4	79.5 ± 0.6
PVP	98.1 ± 0.9	99.0 ± 0.7	98.2 ± 0.7	83.7 ± 0.9	101.3 ± 0.7	101.9 ± 0.4	75.2 ± 0.5	81.1 ± 0.9
Lactose monohydrate	100.3 ± 0.6	101.8 ± 0.6	73.7 ± 0.3	76.1 ± 0.6	98.9 ± 0.9	101.3 ± 0.8	78.3 ± 0.8	74.2 ± 0.8
PEG	99.1 ± 0.7	99.8 ± 0.5	103.1 ± 0.3	98.2 ± 0.9	99.9 ± 0.5	99.1 ± 0.6	82.4 ± 0.7	86.9 ± 0.4
